# Inhibition of Tyrosine Kinase Receptor Tie2 Reverts HCV-Induced Hepatic Stellate Cell Activation

**DOI:** 10.1371/journal.pone.0106958

**Published:** 2014-10-10

**Authors:** Samuel Martín-Vílchez, Yolanda Rodríguez-Muñoz, Rosario López-Rodríguez, Ángel Hernández-Bartolomé, María Jesús Borque-Iñurrita, Francisca Molina-Jiménez, Luisa García-Buey, Ricardo Moreno-Otero, Paloma Sanz-Cameno

**Affiliations:** 1 Department of Cell Biology, University of Virginia School of Medicine, Charlottesville, Virginia, United States of America; 2 Unidad de Hepatología, Hospital Universitario de la Princesa, Instituto de Investigación Sanitaria Princesa (IIS-IP), Madrid, Spain; 3 Centro de Investigación Biomédica en Red de Enfermedades Hepáticas y Digestivas (CIBER-ehd), Instituto de Salud Carlos III (ISCIII), Madrid, Spain; 4 Unidad de Biología Molecular, Hospital Universitario de la Princesa, Instituto de Investigación Sanitaria Princesa (IIS-IP), Madrid, Spain; Rosalind Franklin University of Medicine and Science, United States of America

## Abstract

**Background:**

Hepatitis C virus (HCV) infection is a major cause of chronic liver disease (CLD) and is frequently linked to intrahepatic microvascular disorders. Activation of hepatic stellate cells (HSC) is a central event in liver damage, due to their contribution to hepatic renewal and to the development of fibrosis and hepatocarcinoma. During the progression of CLDs, HSC attempt to restore injured tissue by stimulating repair processes, such as fibrosis and angiogenesis. Because HSC express the key vascular receptor Tie2, among other angiogenic receptors and mediators, we analyzed its involvement in the development of CLD.

**Methods:**

Tie2 expression was monitored in HSC cultures that were exposed to media from HCV-expressing cells (replicons). The effects of Tie2 blockade on HSC activation by either neutralizing antibody or specific signaling inhibitors were also examined.

**Results:**

Media from HCV-replicons enhanced HSC activation and invasion and upregulated Tie2 expression. Notably, the blockade of Tie2 receptor (by a specific neutralizing antibody) or signaling (by selective AKT and MAPK inhibitors) significantly reduced alpha-smooth muscle actin (α-SMA) expression and the invasive potential of HCV-conditioned HSC.

**Conclusions:**

These findings ascribe a novel profibrogenic function to Tie2 receptor in the progression of chronic hepatitis C, highlighting the significance of its dysregulation in the evolution of CLDs and its potential as a novel therapeutic target.

## Introduction

Hepatitis C virus (HCV) infection is a major cause of chronic liver disease (CLD) in developed countries, including chronic hepatitis C (CHC), fibrosis, cirrhosis and hepatocellular carcinoma (HCC) [1], [2]. Unresolved chronic HCV infection triggers the persistent stimulation of immune responses and tissue repair mechanisms, which propel the progression of CHC toward cirrhosis and hepatocarcinoma (HCC) through incessant activation of fibrogenic and angiogenic processes [3], [4], [5].

Liver fibrosis is often observed in chronic HCV infections and is sustained primarily by liver-specific cells, called hepatic stellate cells (HSC). HSC are major injury-sensing cells in the liver, and their overactivation is considered the central event in the development of fibrosis and, ultimately, cirrhosis [6], [7]. Once activated, HSC become highly proliferative and contractile, increase their migratory abilities, and secrete extracellular matrix compounds, such as collagen and extracellular matrix (ECM) proteins [8], [9], [10], [11]. In addition, HSC secrete several growth factors, such as vascular endothelial growth factor (VEGF), connective tissue growth factor (CTGF), and platelet-derived growth factor (PDGF), which promote the differentiation of mesenchymal cells and endothelial activation, migration, and proliferation [6], [12].

This sequence of events effects the accumulation of ECM substances and endothelial and myofibroblast-like cells, which occlude sinusoidal fenestrations, altering the proper interchange of metabolites and oxygen between hepatocytes and blood. This process, termed sinusoidal capillarization, results in increased intrahepatic resistance to blood flow and oxygen delivery, to which HSC respond by increasing their expression of angiogenic factors, such as VEGF and angiopoietin-1 (Ang1), as well as the respective receptors, VEGFR-2 and Tie2, exacerbating the pathology by enhancing cellular proliferation, migration, and deposition of ECM compounds [13].

Neoangiogenesis is a common feature of many CLD [14], [15]; particularly, CHC is notably characterized by the development of an abnormal angioarchitecture in the liver, which is strongly linked with the fibrogenic progression of the disease. Accordingly, considerable alterations in systemic levels of diverse angiogenic factors have been reported in patients with CHC, being angiopoietin 2 (Ang2) significantly related to the fibrosis stage [16], [17]. Due to HSC express angiopoietin's receptor Tie2 [18], a central regulator of physiological and pathological angiogenesis, we aimed to study the fibrogenic role of HCV-infected hepatocytes on HSC activation via Angiopoietin/Tie2 signaling axis. With that aim, we studied the expression of Tie2 receptor throughout the *in vitro* and HCV-induced activation of HSC mainly focused on investigating the effects of Tie2 inhibition on HSC behaviour as potential antifibrogenic target.

Results demonstrated that the tyrosine kinase Tie2 receptor is upregulated during HSC activation. This phenomenon was enhanced by conditioned media from HCV-expressing cells and mediated the activation and migration of HSC. Consistent with these findings, Tie2 blockade by a neutralizing antibody reduced HSC activation with regard to alpha-smooth muscle actin (α-SMA) expression and their migratory and invasive capacity. Inhibition of the key Angiopoietin/Tie2 signaling pathways PI3K/AKT and MAPK [19] notably diminished Tie2 expression on HSC and their activated phenotype. These findings reveal the significance of Tie2 in CHC progression and its related fibrogenesis, highlighting this signaling route as a valuable pharmacological target for CLD intervention.

## Materials and Methods

### Ethics statement

This study was approved by the Ethical Committee of Hospital Universitario de La Princesa and conducted per the Declaration of Helsinki.

### Cell lines and culture conditions

The human hepatic stellate cell line LX-2 [20], plated at 50,000 cells/cm^2^, was grown in Dulbecco's modified Eagle's medium (DMEM) that was supplemented with 10% fetal bovine serum (FBS), 2 mM glutamine, and 100 U/ml penicillin for 24 hours until 100% adherence and shifted to 2% FBS DMEM.

The HCV replicons HCV-C5 and HCV-C7, kindly provided by Dr. Aldabe (Hospital Universitario de Navarra, CIMA), express the complete genome of HCV [21], [22] and were generated in the Huh7 cell line from the genotype 1b HCV strain.

Hepatocyte cell lines were grown in DMEM with 10% FBS, 2 mM glutamine, and 100 U/ml penicillin, and HCV-expressing cells (C5 and C7) were selected specifically with 500 µg/ml G418 (Geneticin; Life Technologies GmbH). When hepatocyte cultures reached 60% confluence, they were shifted to 0% FBS DMEM without G418; the supernatants that were collected at 24, 48, 72, and 96 hours were used as conditioned media (CM) for further experiments.

Before the experiments, HSC were serum-starved in 0% FBS DMEM for 24 hours until their exposure to CM from HCV replicons or Huh7 cells. HSC that were grown in 0% and 10% FBS were used as negative and positive activation controls, respectively.

### Blockade of Tie2 signaling

To determine the Tie2-mediating effects on HSC activation and to analyze the signaling pathways that are involved, 8 µg/ml of neutralizing anti-Tie2 (AF313, R&D Systems, Minneapolis, MN), 8 µg/ml of an isotype control antibody (BD 340473, Becton Dickinson, Mountain View, CA), 25 µmol/mL of PI3-K inhibitor or 25 µmol/mL of MAPK inhibitor (LY294002 and PD98059, respectively, Calbiochem, La Jolla, CA) were added to the appropriate cell cultures.

### Western blot and zymography

Whole HCV replicons and HSC extracts were obtained with Laemmli buffer and sonicated (Soniprep 150, MSE, UK); the insoluble debris was removed by centrifugation at 9500 *g* for 5 minutes. Total protein extracts were resolved by SDS-PAGE and transferred to a nitrocellulose membrane (Bio-Rad). The membranes were blocked with a 7.5% solution of nonfat dry milk in Tris-HCl-buffered solution (TBS, pH 7.5) and incubated with antibodies against COL-I (H-197, Santa Cruz Biotechnology, Santa Cruz, CA, at 1∶2000), Tie2 (AF313, R&D Systems, 1∶200), matrix metalloproteinase-2 (AF902, R&D Systems, 0.2 µg/mL), α-SMA, (1A4, Sigma-Aldrich, St. Louis, MO, 1∶1000), Tubulin (DM1A, 1∶500, Sigma-Aldrich, St. Louis, MO, 1∶5000), HCV core (C7–50, Santa Cruz Biotechnology, Santa Cruz, CA, 1∶500) or NS5A (6F3, ViroStat, Portland, ME, 1∶1000).

After extensive washes, the membranes were incubated with blocking buffer, containing horseradish peroxidase-conjugated goat anti-mouse or goat anti-rabbit (Santa Cruz Biotechnology, Santa Cruz, CA) at 1∶1000. Proteins were detected by chemoluminescence (SuperSignal West Pico, Thermo Fisher Scientific Inc, Rockford, IL USA), and band intensities were quantitated using FUJIFILM Science Lab Image Gauge Ver. 4.0; expression of proteins was normalized to that of Tubulin for each lysate. Zymographies were performed essentially as described [23] by separating equal amounts of total HSC extracts on a 10% polyacrylamide gel that contained 1% gelatin.

### Cell migration assay

The migratory and invasive abilities of HCS were analyzed in a 24-well transwell, containing 8-µm pore inserts (Corning, Corning, NY), that was precoated with collagen (Pure Col, Advanced BioMatrix, California, EE.UU.). CM from Huh7 cells or HCV-derived replicons (600 µL) were placed in the bottom compartment of the chamber. After overnight serum starvation, 100 µL of LX-2 cell suspension (500 cells/µL) was added to the upper compartment. The chambers were incubated at 37°C with 5% CO2 for 24 hours. Then, the culture medium was removed, and adherent cells were fixed with 4% paraformaldehyde and stained with DAPI solution. The number of nuclei from LX-2 cells on the lower surface of the filters was counted in 5 randomly chosen microscopic fields for each specimen on an epifluorescence Leica microscope (Leica, Wetzlar, Germany).

### Statistical analysis

Data were expressed as mean ± standard deviation (SD). Statistical analysis of the results was performed using unpaired student's *t* test. Two-tailed P values below 0.05 were considered significant (SPSS 16.0, SPSS, Chicago, IL).

## Results

### Tie2 expression is upregulated in HSC cultures in a time-dependent manner

**Figure 1:**
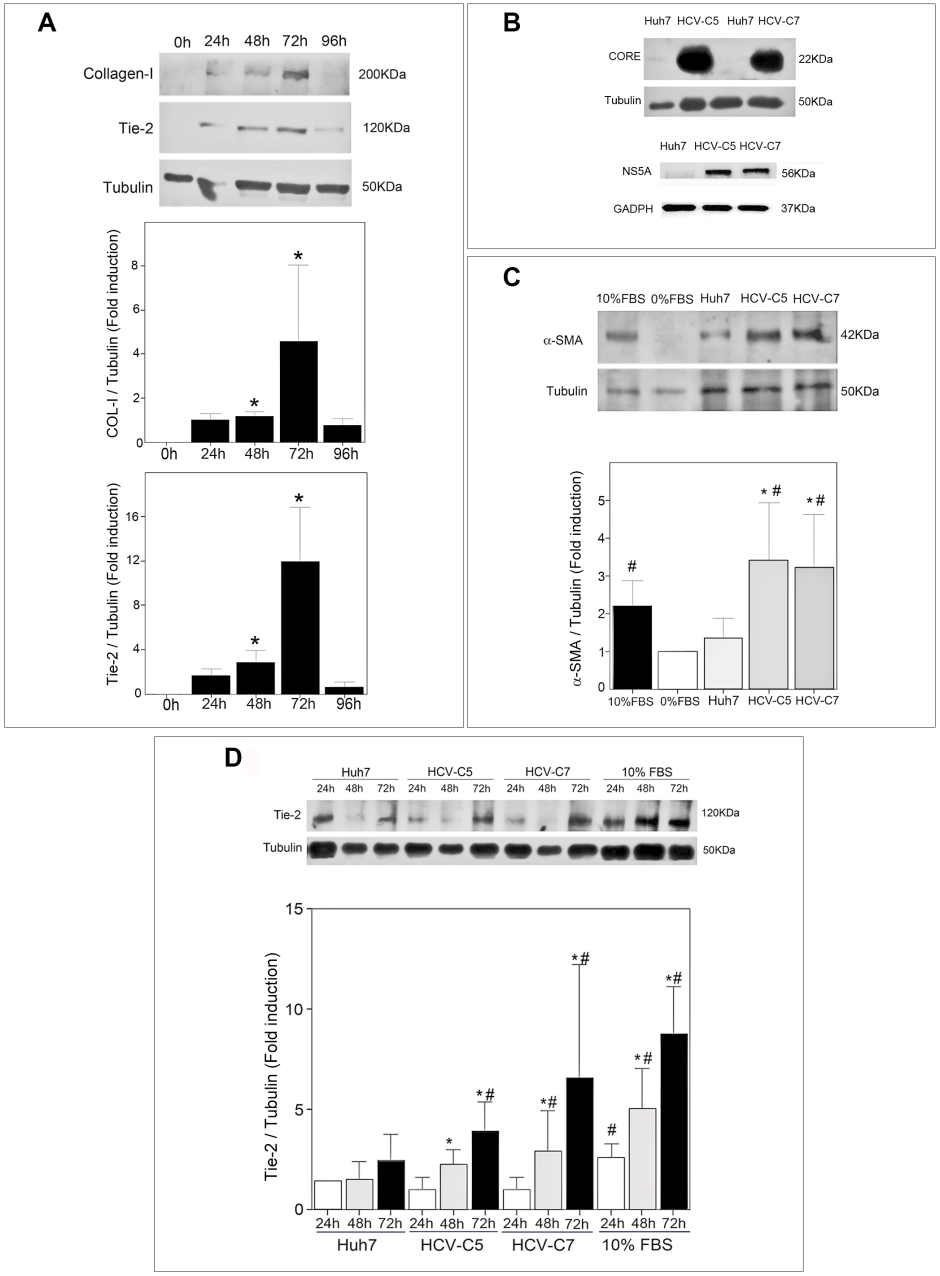
Activation of HSC increases Tie2 expression. (A) Kinetics of Tie2 expression during *in vitro* activation of HSC. HSC, plated at 50,000 cells/cm2 density and grown in 2% DMEM during 24, 48, 72 and 96 hours, were lysed in Laemmli buffer and loaded in 7% acrylamide gels (20 µL of total protein extract per well). SDS-PAGE resolved proteins were transferred to nitrocellulose membranes and probed with respective antibodies against COL-I (H-197, 1∶2000), Tie2 (AF313, 1∶200) or tubulin (DM1A, 1∶5000). Quantitative analysis of Tie2 or COL-1 chemiluminescence in relation to tubulin (FUJIFILM Science Lab Image Gauge) is shown in respective graphs. *p<0.05 *versus* 24 h (mean +SD, 3 independent experiments). (B) Expression of HCV proteins by hepatic cell lines (Huh7 or HCV replicons). Protein lysates from hepatic cells (20 µL each) were SDS-PAGE resolved and probed with antibodies against Core (C7–50, 1∶500), NS5A (6F3, 1∶1000) or tubulin (DM1A, 1∶5000). (C) Expression of α-SMA in HSC exposed to conditioned media (CM) from hepatic cell lines (Huh7 and HCV replicons) compared to that expressed by HSC incubated with 0% FBS DMEM. Expression α-SMA by HSC grown in 10% FBS DMEM was used as positive control of HSC activation. CM from hepatic cells, plated at equal densities and cultured during 24 h in 0% FBS DMEM, were used to grow HSC deprived of serum 24 h before. After HSC lysis in Laemmli, equal volumes (20 µL) of protein extracts were analyzed for α-SMA expression by western blotting (1A4, 1∶1000). Tubulin expression (DM1A, 1∶5000) was also evaluated in order to control protein loading and relative α-SMA/tubulin data are shown in the graph (mean +SD of 3 independent experiments). #p<0.05: statistic differences *versus* cultures in 0% FBS DMEM; *p<0.05: statistic differences *versus* Huh7. (D) HSC conditioned during different times with media from hepatic cells (Huh7 or HCV replicons) or 10% FBS DMEM. HSC, plated at same density in 0% FBS DMEM 24 h before, were exposed during 24, 48 or 72 h to 10% FBS DMEM or CM from hepatic cell lines and the expression of Tie2 (AF313, 1∶200), normalized with tubulin (DM1A, 1∶5000), was analyzed. Graph shows quantitative densitometric analysis of western blot bands (mean +SD of 3 independent experiments). #p<0.05: statistic differences *versus* Huh7 at same times; *p<0.05, same CM *versus* 24 h.

During activation of HSC by culture, the expression of Tie2 increased progressively, peaking at 72 hours of incubation. As Figure 1A shows, the rise in Tie2 was linked to the enhancement of COL-I, a key marker of HSC activation (p<0.05). Further, the upregulation of these molecules correlated significantly at 48 and 72 hours (data not shown).

### HCV replicons activate HSC

HCV-C5 and HCV-C7, previously characterized as described [22], were grown in serum-starved media at various times and used to obtain CM. The expression of viral proteins in HCV-expressing hepatocytes was confirmed by assessing the expression of core and NS5A proteins in the lysates of HCV replicons (Figure 1B). As figure 1C shows, CM from HCV-expressing cells upregulated the activation marker α-SMA in HSC compared with CM from Huh7.

In addition, Tie2 expression rose on HSC that were exposed to HCV-C5 and HCV-C7 CM in contrast to HSC that were conditioned with CM from Huh7 cells (Figure 1D, p<0.05). CM from HCV replicons increased Tie2 expression on HSC at 48 and 72 hours compared with 24 hours (Figure 1D, p<0.05). However, HSC failed to significantly enhance Tie2 expression when exposed to CM from Huh7 cells at any time.

### HCV replicons promote HSC invasion

**Figure 2:**
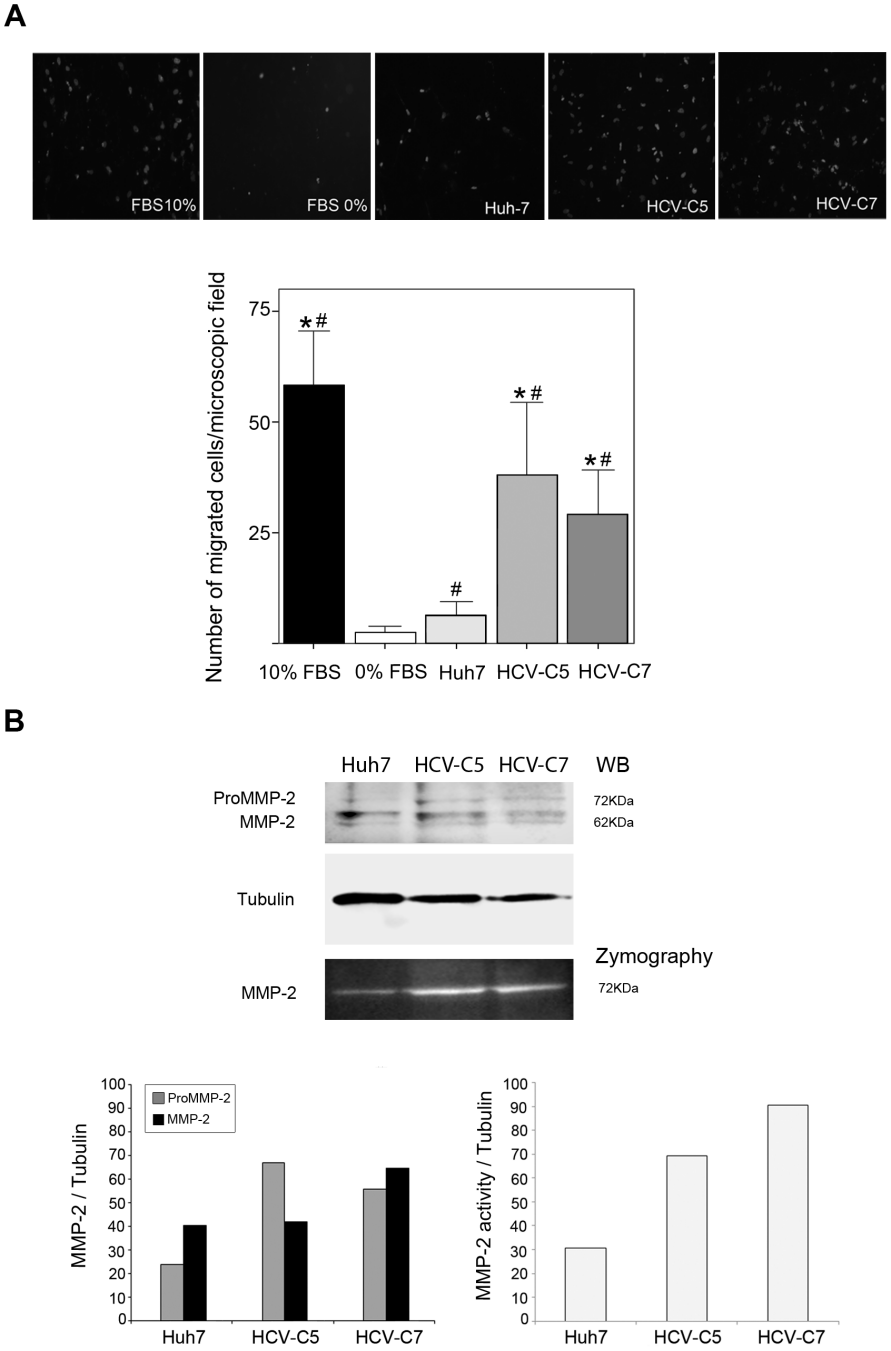
Conditioned media from HCV-expressing cells enhance invasive potential of HSC. (A) Effect of different CM on HSC invasion through transwell inserts precoated with collagen. Same amount of serum starved HSC (5×10^4^ cells/100 µL) were seeded at the upper chambers of transwells and exposed during 24 h to different CM (hepatic-derived CM, 0% FBS DMEM or 10% FBS DMEM) dispensed at the lower compartments. At the end of migration, the upper surface of the membrane was washed and HSC adhered to the lower surface were fixed in methanol, stained with DAPI and counted in 5 randomly chosen microscopic fields (400x) in an epifluorescence microscope (Leica, Wetzlar, Germany). Data from 4 independent experiments are shown as mean +SD. #p<0.05, HSC cultured with CM from HCV replicons or DMEM 10% FBS *versus* HSC cultured in DMEM 0% FBS; *p<0.05, HSC cultured with CM from HCV-expressing cells or 10% FBS *versus* HSC cultured with CM from Huh7. (B) ProMMP-2 and MMP-2 expression (AF902, 0.2 µg/mL) and activity were respectively examined by western blotting (WB) and zymography and further quantified in protein extracts (20 µL) of HSC cultured during 24 h with CM from Huh7 or HCV-expressing cells. Bars represent the mean of densitometric analysis from two independent experiments.

HCV replicons significantly stimulated the invasive potential of HSC. As Figure 2A illustrates, HSC that were exposed to HCV-C5 or HCV-C7 CM enhanced their migration and invaded the collagen matrix to a greater extent than HSC that were cocultured with Huh7 or 0% FBS control media (Figure 2A, p<0.05 all). Notably, the invasion of HSC that were exposed to media from HCV replicons was accompanied by an increase in MMP-2 expression and activity, as shown by western blot and zymography (Figure 2B). However, this effect was not so evident in HSC that were exposed to CM from Huh7 cells.

### Effect of Tie2 blockade on HSC physiology

**Figure 3:**
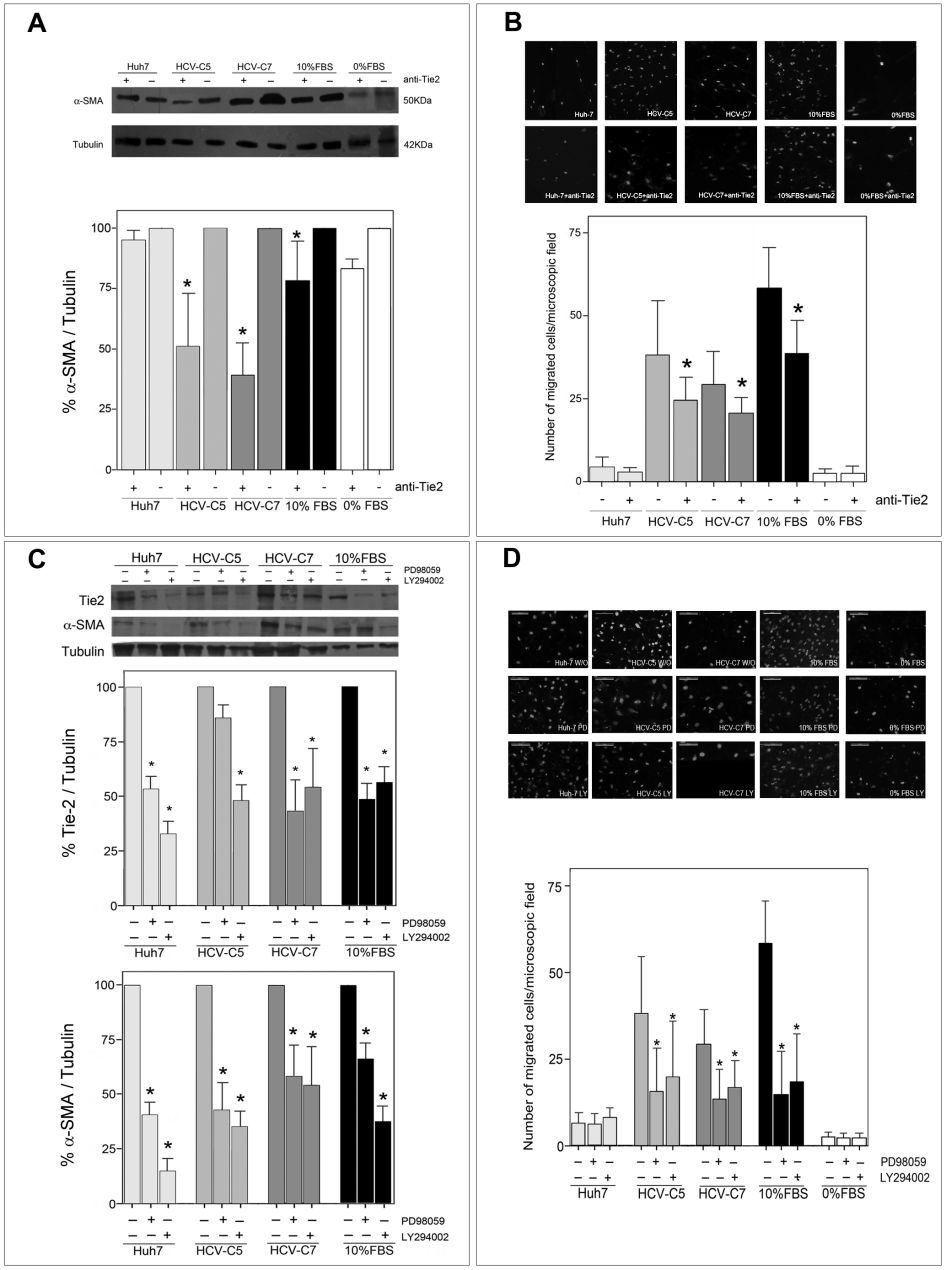
The activation and invasive potential of HSC are prevented by a Tie2 neutralizing antibody or by the inhibition of Akt/PI3k and MAPK signaling pathways. (A) α-SMA expression was assessed by western blotting (1A4, 1∶1000) in 20 µL of Laemmli lysates from HSC exposed during 24 h to different culture conditions (hepatic-derived CM, 0% FBS DMEM or 10% FBS DMEM) in presence (+) or absence (-) anti-Tie2 blocking antibody (AF313, 8 µg/ml). Quantitative analysis of α-SMA/tubulin bands in presence of neutralizing antibody is displayed in the graph as percentage of expression observed without anti-Tie2 (100%) for each experimental condition. Bars show mean +SD of 3 independent experiments. *p<0.05. (B) HSC cells (5×10^4^ cells/100 µL) cultured on upper transwell chambers able to invade collagen under different stimuli (hepatic-derived CM, 0% FBS DMEM or 10% FBS DMEM) with or without AF313 anti-Tie2 antibody (8 µg/ml) dispensed at bottom compartments of transwell are illustrated in the representative epifluorescence pictures after DAPI staining (400x). 5 randomly selected microscope fields were quantified per experiment (3 independent). Bars show the mean +SD average of migrating cells per field from the different experiments. *p<0.05 indicates statistical differences owing to the presence of anti-Tie2 neutralizing antibody. (C) The influence of different CM in presence or absence of LY294002 and PD98059 at 25 µmol/ml each (Akt/PI3k and MAPK inhibitors, respectively) on the expression of Tie2 (AF313, 1∶200) and α-SMA (1A4, 1∶1000) by HSC is illustrated. Respective western blots were analyzed in relation to tubulin to normalize total protein loading and the expression in presence of inhibitors was displayed as percentage of the expression without the respective compound (100%) in the same experimental conditions. Data from 3 experiments in duplicate are shown. *p<0.05. (D) Invasive potential of HSC (5×10^4^ cells/100 µL) under the influence of LY294002 and PD98059 (25 µmol/ml both) at different experimental conditions (hepatic-derived CM, 0% FBS DMEM or 10% FBS DMEM) is shown by fluorescence images (400x). Graph depicts the mean +SD of average migrating HSC/field (5 microscope fields) per experiment (3 independent) of denoted HSC culture conditions. *p<0.05: statistical difference of presence *versus* absence of inhibitors.

Pretreatment of HSC with the specific Tie2-neutralizing antibody (AF313, R&D Systems, Minneapolis, MN) downregulated α-SMA expression, as shown in Figure 3A, but this blockade was not effective in HSC that were conditioned with media from Huh7 cultures. This effect was not observed when a control isotype antibody was added instead anti-Tie2 neutralizing antibody (Figure S1), which argues in favour of Tie2 specificity. Similarly, Tie2 blockade by the same neutralizing antibody markedly reduced the invasion of HSC that were conditioned with media from HCV-C5 and HCV-C7 replicons through transwell inserts (Figure 3B).

Additionally, because PI3K/AKT and MAPK signaling mediates the key effects of angiopoietin/Tie2, including a wide range of downstream targets that regulate tumor-associated processes, such as cell growth, cell cycle progression, survival, migration, epithelial-mesenchymal transition, and angiogenesis, we examined the influence of selective PI3K/AKT and MAPK neutralization on Tie2-mediated HSC activation and migration. Administration of PI3-K/AKT or MAPK inhibitors (LY294002 and PD98059, respectively) to HSC cultures that were activated with CM from HCV replicons downregulated α-SMA and Tie2 expression (Figure 3C), which was followed by a notable decrease in their migration rate (Figure 3D).

## Discussion

Liver fibrosis and angiogenesis have been suggested to contribute significantly to the progression of CLD [8], [24]. Earlier results from our group and other laboratories have reported a link between the dysregulation of intrahepatic vascular homeostasis and the stage of liver disease. Further, the upregulation of several angiogenic mediators, such as Ang2 and VEGF, in diverse chronic inflammatory diseases and CLD [15], [25], [26], [27], [28], [29] prompted us to implicate them as noninvasive surrogate markers of CHC evolution [16], [17], [30].

In chronic liver injury, the self-limiting inflammation, necrosis, and regeneration of the hepatic parenchyma are perturbed by the persistence of injury. Quiescent HSC experience constant activation, which entails significant phenotypic and functional changes in cultured human and rat HSC [6], [31]. These changes, comprising the acquisition of a contractile, proliferative, and profibrogenic phenotype by HSC, effect the overproduction of ECM compounds, which ultimately results in architectural and functional alterations of the liver [9], [11], [12], [32], [33], [34], [35].

Angiogenesis is also stimulated during fibrotic development in an attempt to restore the intrahepatic-blood interchange of cells and metabolites that is impaired by the progressive sinusoidal deposition of ECM. The fibrotic background promotes the upregulation of MCP1, VEGF, and Ang1 by HSC in response to surrounding stimuli (eg, hypoxia and leptins) [13], [36], [37], [38], accelerating the development of a disorganized and dysfunctional intrahepatic vasculature [3].

Consistent with the upregulation of the VEGF receptors Flt-1 and Flk-1 and Tie2 by activated HSC in areas of active fibrogenesis [18], [38], we noted time-dependent upregulation of Tie2 during *in vitro* HSC activation.

Despite previous studies have pointed out some discrepancies concerning the potential paracrine effects of hepatic cells expressing HCV proteins on HSC physiology, main results from stable hepatic transfectants of HCV proteins, HCV genomic and subgenomic replicons or infectious JFH1 HCV cultures, account for the substantial profibrogenic effects of HCV-expressing cells towards the activation of HSC. It has been described that recombinant core [39], as well as core-expressing cells, stimulate HSC activation as detected by the augmented expression of α-SMA [40], [41]. In addition, nonstructural genes of HCV promote the expression of profibrogenic factors by hepatic cells leading to progression of liver fibrosis [42]. HCV core protein may assist hepatic fibrogenesis via up-regulation of CTGF and TGF-beta1 [39]. In addition, comparable significant increases of CTGF and TGF-β1 in a stable E2-expressing Huh7 cell line were also observed [43]. Conversely, the subgenomic replicon expressing HCV nonstructural proteins (NS3-5B) in Huh7 cells did not show considerable effects on proliferation and migration of HSC [44] which suggests higher paracrine effects of HCV structural proteins on such events. However, a differential regulation of core and nonstructural proteins on HSC biologic functions has been reported: whereas the expression of core protein increases cell proliferation in a Ras/ERK and PI3K/AKT dependent manner, NS3-5B protein expression mainly induce proinflammatory processes through the NF-kappa B and c-Jun N-terminal kinase pathways [40]. All these findings indicate both direct and indirect actions of HCV proteins on different aspects of HSC physiology, finally contributing to HCV-induced liver fibrosis.

In addition, HSC that were exposed to HCV replicons became highly invasive and had greater MMP2 expression and activity. Core and nonstructural HCV proteins affect a wide range of hepatocyte processes such as proliferation, adhesion, autophagy, and chemokine secretion [45], [46], [47], regulating the fibrogenic properties of HSC [48], [49], [50]. Further, HCV RNA replication alters the expression of extracellular matrix-related molecules in HSC [50]. These findings demonstrate that certain factors that are released by HCV-infected hepatocytes modify HSC behavior, enhancing their migration and enzymatic activities, ultimately effecting the fibrogenic progression of CHC.

Despite fibrogenic effects of HCV-expressing hepatocytes via TGF-β1 have been established, the observed induction of Tie2 throughout HSC activation suggests the possible paracrine role of Angiopoietins/Tie2 axis on liver fibrogenesis. Accordingly, it has been described that HBV/HCV could enhance Ang2 promoter expression through mitogen-activated protein kinase (MAPK) pathways [51] and other authors have reported that pericytes express a functionally active Tie2 receptor which may be significantly upregulated by both Ang1 and Ang2, concluding that the Angiopoietin/Tie2 axis influences the activation state and recruitment of pericytes during angiogenesis [52]. Hence, these findings might indicate the relevant role of Tie2 receptor on HCV-induced fibrogenesis. However, the molecular basis by which HCV affects Ang/Tie2 signaling requires further investigation.

In addition, we noted that blockade of Tie2 signaling by a specific neutralizing antibody significantly reduced α-SMA expression, primarily on activated HSC (exposed to 10% FCS and HCV replicon CM), impairing their invasive potential. Our finding that Tie2 blockade counteracts the activation of HSC by HCV replicons suggests that Tie2 signaling is critical in promoting and sustaining the profibrotic features of HSC during HCV infection.

As described, Tie2 receptor primarily involves the PI3K/Akt and MAPK signaling—essential pathways that have been implicated in cell survival, migration, and invasion [19], [53],[54],[55] and modulates important functions in HSC [19], [56], [57], [58], [59].

Accordingly, blockade of the Akt/PI3k and MAPK pathways by selective inhibitors decreased the activation and migration of HSC, accompanied by the downregulation of Tie2. These findings suggest a regulatory feedback loop of Tie2 signaling that modulates HSC functions.

Paradoxically, VEGF-induced shedding of Tie2 by AKT signaling has been recently described in endothelial cells, and AKT-induced Ang2 promotes the endocytosis of Tie2 [60], [61], [62]. Yet, like other RTK receptors, Tie2 regulation is cell type- and context-dependent and tightly regulated over time and by location; thus, depending on cell type and conditions (resting or stimulated), Tie2 interacts with various coreceptors (integrins, Tie1) and ligands (Ang1 and Ang2, at different multimeric orders), leading to many effects [63], [64], [65]. Consequently, the modulation of cell-matrix interactions by Tie2 ligand (Ang1/Ang2) or Tie2-coreceptor complexes (Tie1, integrins), based on subcellular location, effects many responses by altering localized signaling routes [66], [67], explaining the conditional agonistic *versus* antagonistic function of Ang2. Thus, the effects of HCV on HSC physiology implicate a novel profibrogenic mechanism that promotes the progression of CHC via paracrine upregulation of Tie2.

The profibrogenic and proinflammatory nature of activated HSC has been characterized extensively, but their function in pathological angiogenesis remains poorly understood. HSC are an important source of angiogenic cytokines under hypoxia [2], [13], [38] and in acute and chronic liver injury [2], [15], [68], [69], [70]. In addition, recent studies have identified HSC as important targets of VEGF and Ang1, which stimulate their proliferation, chemotaxis, and synthesis of type I collagen [69], [70]. These data, with our findings regarding the implication of Tie2 receptor in modulating HSC function by HCV replicons, suggest that the dysregulation of Tie2 signaling orchestrates key events in HSC physiology, leading to CHC pathogenesis through neoangiogenesis, inflammation, and fibrogenesis.

**Figure 4:**
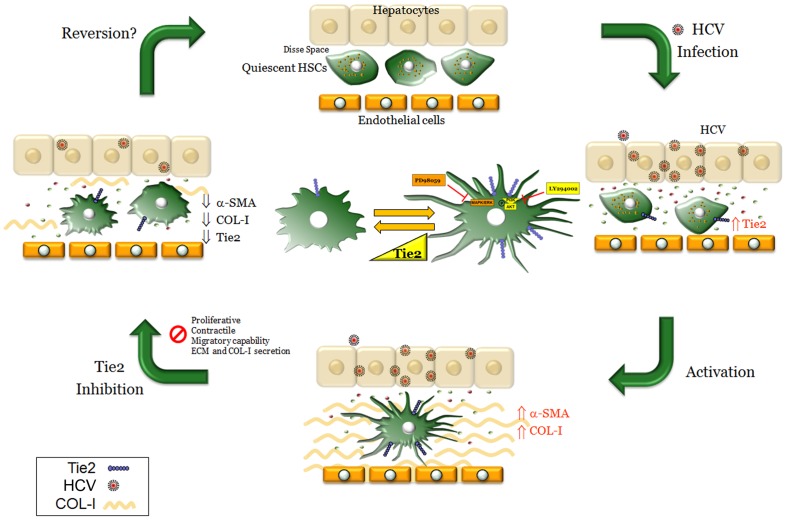
Phenotypic and functional changes in HSC during chronic HCV infection. After liver injury, hepatic stellate cells undergo activation, which entails the transition of quiescent cells into proliferative, fibrogenic, and contractile myofibroblasts. HCV-infected hepatocytes release factors that induce paracrine activation of HSC, triggering the expression of angiogenic Tie2 receptor, COL-I, and α-SMA and their invasive and migratory capacity. Blockade of Tie2 receptor or Akt/PI3k and MAPK signaling reverts the fibrogenic features of HSC during HCV infection. This phenomenon might facilitate the regression to quiescent HSC, raising the possibility of resolution of liver injury.

Inhibition of Tie2 signaling might have antifibrotic effects through impaired activation and recruitment of these fibrogenic cells to injured tissues, preventing the release and accumulation of ECM compounds and their pathological effects on sinusoidal architecture and physiology (Figure 4). Thus, the reversion of HSC activation by inhibition of Tie2 signaling implicates the angiopoietin-Tie2 axis as a potential therapeutic target.

In summary, our results demonstrate that factors that are released by HCV-expressing hepatocytes effect the acquisition of an invasive profibrotic phenotype in HSC via the Tie2 receptor. Notably, Tie2 receptor blockade and Akt/PI3k and MAPK inhibitors significantly impair crucial fibrogenic events that are induced by HCV, such as HSC activation and migration. Our data indicate that the angiogenic Tie2 receptor regulates HSC physiology in HCV infection. An in-depth study of the complex regulation of Tie2 should increase our understanding of the molecular mechanisms of the progression of CLD, providing novel and valuable therapeutic strategies for antifibrotic pharmacological interventions.

## Supporting Information

Figure S1 Anti-Tie2 neutralizing antibody reduces HSC activation. The expression of α-SMA by HSC exposed during 24 h to different conditioned media (0% FBS DMEM, Huh7, HCV-C5, HCV-C7 and 10% FBS DMEM) was assessed by western blotting in presence (+) or absence (−) of anti-Tie2 blocking antibody (AF313, 8 µg/ml) or isotype control antibody (BD-340473, 8 µg/ml). Quantitative analysis of α-SMA/GAPDH bands for each experimental condition is displayed in the graph. Bars show the mean of 2 independent experiments.
(TIF)
