# Supplementary figures and images for: Inhibition of Tyrosine Kinase Receptor Tie2 Reverts HCV-Induced Hepatic Stellate Cell Activation
Source: PLoS One. 2014 Oct 10;9(10):e106958. doi: 10.1371/journal.pone.0106958 (PMC4193738; doi:10.1371/journal.pone.0106958)

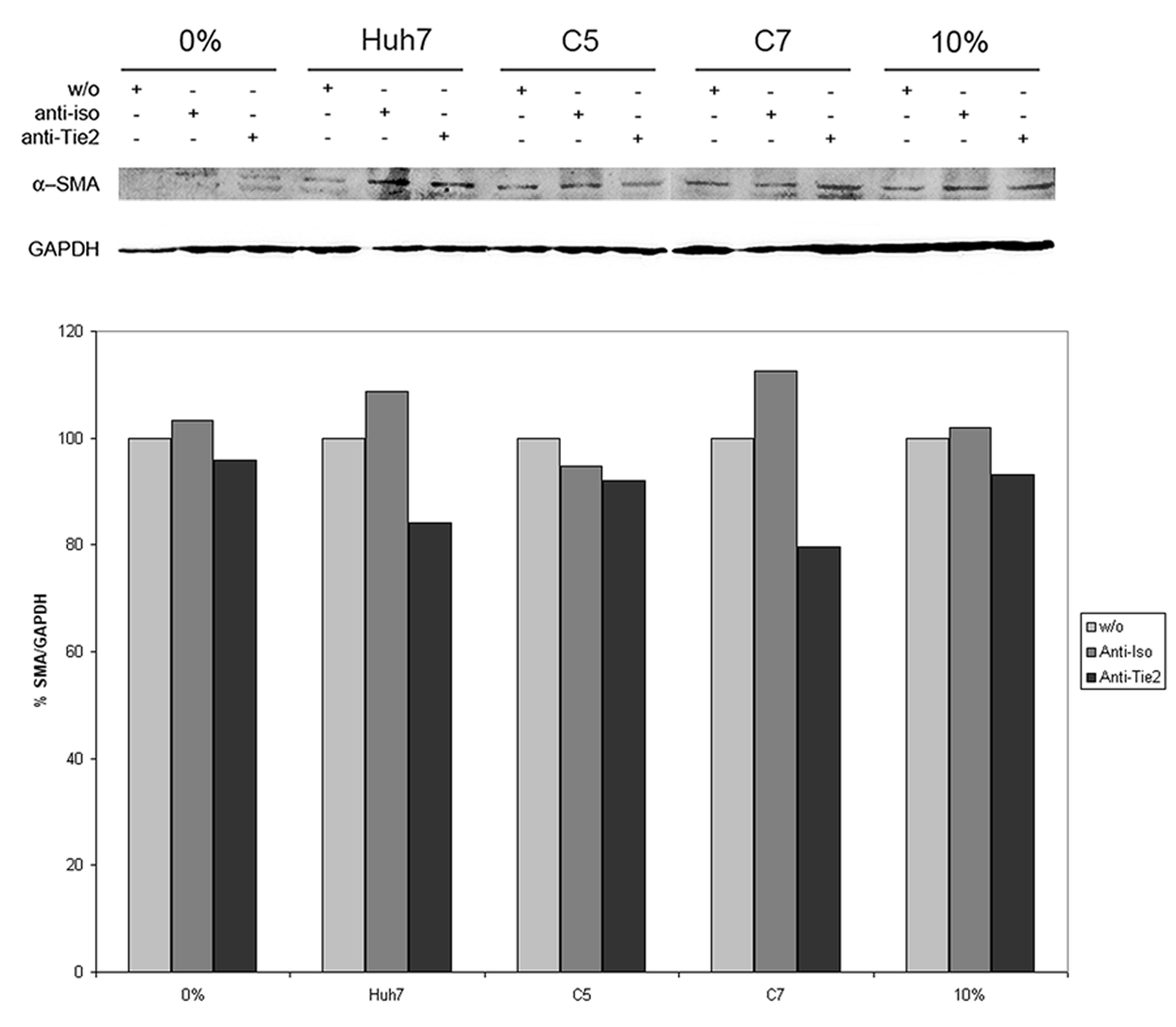

Supplement: Figure S1 — Anti-Tie2 neutralizing antibody reduces HSC activation. The expression of α-SMA by HSC exposed during 24 h to different conditioned media (0% FBS DMEM, Huh7, HCV-C5, HCV-C7 and 10% FBS DMEM) was assessed by western blotting in presence (+) or absence (−) of anti-Tie2 blocking antibody (AF313, 8 µg/ml) or isotype control antibody (BD-340473, 8 µg/ml). Quantitative analysis of α-SMA/GAPDH bands for each experimental condition is displayed in the graph. Bars show the mean of 2 independent experiments. (TIF) [file pone.0106958.s001.tif]
